# Best Evidence Rehabilitation for Chronic Pain Part 5: Osteoarthritis

**DOI:** 10.3390/jcm8111769

**Published:** 2019-10-24

**Authors:** David Rice, Peter McNair, Eva Huysmans, Janelle Letzen, Patrick Finan

**Affiliations:** 1 Health and Rehabilitation Research Institute, Auckland University of Technology, Auckland 1142, New Zealand; peter.mcnair@aut.ac.nz; 2 Waitemata Pain Service, Department of Anaesthesiology and Perioperative Medicine, Waitemata District Health Board, Auckland 1142, New Zealand; 3 Pain in Motion International Research Group; Eva.Huysmans@vub.be; 4 Department of Physiotherapy, Human Physiology and Anatomy, Faculty of Physical Education & Physiotherapy, Vrije Universiteit Brussel, 1090 Brussel, Belgium; 5 Department of Public Health (GEWE), Faculty of Medicine and Pharmacy, Vrije Universiteit Brussel, 1090 Brussels, Belgium; 6 I-CHER, Interuniversity Center for Health Economics Research, 1090 Brussels, Belgium; 7 Department of Physical Medicine and Physiotherapy, Universitair Ziekenhuis Brussel, 1090 Brussels, Belgium; 8 Department of Psychiatry & Behavioral Sciences, Johns Hopkins University, Baltimore, MD 21287, USA; jletzen1@jhmi.edu (J.L.); pfinan1@jhu.edu (P.F.)

**Keywords:** osteoarthritis, musculoskeletal pain, rehabilitation medicine, physiotherapy, psychology, non-pharmacological

## Abstract

Osteoarthritis (OA) is a leading cause of chronic pain and disability in older adults, which most commonly affects the joints of the knee, hip, and hand. To date, there are no established disease modifying interventions that can halt or reverse OA progression. Therefore, treatment is focused on alleviating pain and maintaining or improving physical and psychological function. Rehabilitation is widely recommended as first-line treatment for OA as, in many cases, it is safer and more effective than the best-established pharmacological interventions. In this article, we describe the presentation of OA pain and give an overview of its peripheral and central mechanisms. We then provide a state-of-the-art review of rehabilitation for OA pain—including self-management programs, exercise, weight loss, cognitive behavioral therapy, adjunct therapies, and the use of aids and devices. Next, we explore several promising directions for clinical practice, including novel education strategies to target unhelpful illness and treatment beliefs, methods to enhance the efficacy of exercise interventions, and innovative, brain-directed treatments. Finally, we discuss potential future research in areas, such as treatment adherence and personalized rehabilitation for OA pain.

## 1. Introduction 

Osteoarthritis (OA) is the most common form of arthritis and a leading cause of chronic pain and disability, affecting ~250 million people worldwide [1]. OA can occur in any synovial joint, but the knee, hip, and joints of the hand are most commonly affected [2,3]. Important risk factors for the development of OA include increasing age, female gender, previous joint trauma, and (as yet largely unidentified) genetic factors [2,4]. In addition, increased mechanical stress on the joints caused by factors, such as malalignment [2], increased body weight [5,6], and manual work [7,8,9], also play an important role. While the signature characteristic of OA is a loss of articular cartilage, it is apparent that many other joint structures can become affected as the disease progresses, including the subchondral bone, fibrocartilage, capsule, ligaments, synovial membrane, and periarticular muscles [10].

### 1.1. OA Pain Presentation

Pain is the primary symptom that motivates people with OA to seek medical attention and it is associated with functional limitations [11,12,13], emotional distress [14,15], fear of movement [16,17,18], sleep problems [15,19], fatigue [15,20], and an overall marked reduction in quality of life [21,22]. Joint pain might also have direct neuromuscular consequences, including muscle weakness [23], impaired muscle force control [24], and gait adaptations [25], some of which may affect joint loading [26,27] and increase the risk of further pain and structural deterioration [28,29,30]. 

Individuals with symptomatic OA commonly report pain in response to activities of daily living that involve movement or mechanical loading of the affected joint, such as walking across the room, getting up from sitting, or opening a jar [31]. Pain at rest and night pain are also frequently reported [31]. The painful joint(s) is commonly described as more sensitive to touch and pressure [32] and in some cases, changes in temperature [33,34]. Two distinct types of joint pain are commonly reported—a dull background aching, throbbing pain, and a sharp, stabbing pain that is intermittent but more severe [31,35]. In knee OA, specifically, this intermittent, sharp pain often arises unpredictably and is associated with giving way or locking of the knee [31]. A minority of people with OA [36,37,38] describe pain qualities, such as burning, shooting, or electric shocks, and more recent evidence shows that some describe perceptual disturbances, including feeling as if their painful limb is altered in size [39,40,41], missing [32,42], or difficult to control [39,42]. 

OA pain is often described as highly variable, fluctuating in intensity both within and between days [43,44,45]. In the long-term, the natural course of OA pain also varies across individuals. When reassessed over several months or years, many people with OA (35–60%) report a more or less consistent joint pain that does not markedly change over time [46,47,48]. For others, pain is described as consistently worsening, progressing from a predominantly load-dependent intermittent pattern of pain, to a more constant, severe pain [35,49]. Conversely, ~12–30% of people report sustained lessening of pain intensity over several years [47,50,51]. Thus, although OA has traditionally been thought of as a progressive condition, the evidence suggests that long-term worsening of pain is far from inevitable. 

### 1.2. Mechanisms of OA Pain

Historically, OA pain has been viewed as a symptom, being driven by the activation of articular nociceptors in response to structural damage of the joint [52,53]. While joint nociception is one important factor contributing to OA pain, interdisciplinary research has revealed that OA pain is better understood within a biopsychosocial framework [54,55,56]—being influenced by a complex array of interacting factors. 

#### 1.2.1. Peripheral Mechanisms of OA Pain

Notably, articular cartilage is aneural, and therefore cannot generate nociception [57]. In contrast, other joint structures, such as the subchondral bone, periosteum, ligaments, capsule, synovium, and parts of the meniscus are richly innervated by nociceptors [58,59]. Despite this, demonstrating a strong link between joint structural deterioration and OA pain has proven to be elusive. For the individual person with OA, there does not appear to be a meaningful relationship between the structural changes that were observed on x-ray and the intensity of the pain experience [60,61]. At a population level, the relationship is somewhat stronger, with those who have severe radiographic OA more likely to experience frequent pain [57,62,63]. However, as many as 75–80% of community dwelling adults with evidence of radiographic OA do not experience frequent joint pain [64,65], while, conversely, as few as 10–15% of people who experience frequent joint pain have definite radiographic evidence of OA [60,64,65]. Furthermore, the radiographic progression of OA can be discordant with changes in joint pain [48,66]. 

The relatively poor relationship between radiographic findings and OA pain can be partly explained by the lack of sensitivity of x-ray to joint structural changes, particularly in soft tissue and subchondral bone [56,67]. Thus, several studies have examined the relationship between joint MRI features and OA pain. In recent systematic reviews of studies involving people with radiographically established knee OA [68,69], two MRI findings have been consistently associated with both incident joint pain and worsening OA pain—bone marrow lesions (BMLs) and synovitis. BMLs are ill-defined, hyperintense marrow signals on fluid-sensitive, fat-suppressed MRI [70,71]. While still unclear, it is thought that BMLs may trigger nociception through microfracture of the subchondral bone, an increase in intraosseous pressure and/or neoinnervation accompanying vascular in growth [71,72,73]. With respect to synovitis, several inflammatory molecules directly activate chemosensitive nociceptors in the joint, while others also produce potent, long lasting decreases in the firing threshold of nociceptors and increase their spontaneous discharge, a process that is known as peripheral sensitization [58,59]. Thus, synovitis, and the accompanying peripheral sensitization, may substantially increase joint nociceptor discharge, both at rest and during movement. While once considered a non-inflammatory condition, there is now compelling evidence that synovitis is a common feature of OA [74]. 

#### 1.2.2. Central Mechanisms of OA Pain

Despite advances in our understanding of important sources of joint nociception, it is evident that joint MRI features can also be discordant with pain. For example, at least one abnormal joint MRI feature can be found in >80% of pain-free community dwelling adults [67]. Notably, MRI identified synovitis might be present in ~30–35% of pain-free individuals [67,75], while BMLs may be observed in ~30–50% of people who are pain free [75,76,77] and, even in those with established OA, progression or resolution of synovitis [78] and BMLs [79] are not always related to changes in pain. These findings suggest that other factors also play an important role in determining individual differences in OA pain severity. In this regard, extensive preclinical evidence exists that, in the presence of ongoing joint nociception, a maladaptive gain of neural signaling in the central nociceptive pathways within the spinal cord and brain occurs in animal models of arthritis [80,81,82,83], a process that is known as central sensitization. Importantly, central sensitization results in sensory input being strongly amplified when it reaches nociceptive pathways at the level of the spinal cord and brain [84], thereby increasing the frequency, severity, and spread of pain [85,86,87]. In recent decades, mounting evidence from human studies suggests that central sensitization is evident in at least a subgroup of people with OA [88], is an important driver of pain severity [85,87], and at least partly explains the discordance between pain intensity and joint structural changes [89]. In addition, several neuroimaging studies have now demonstrated altered brain structure and brain activation patterns in people with symptomatic OA. Commonly, limbic areas of the brain are more active in people with OA than in controls [90,91,92,93,94], both at rest and in response to standardized painful stimuli. In addition, changes in gray matter volume [93,95,96] and white matter integrity [96] have been shown in several brain regions important to nociceptive processing. 

It is now well established that psychosocial and lifestyle factors (e.g., sleep) play an important role in amplifying or attenuating the pain experience [97,98], and may be involved in the initiation and maintenance of central sensitization [98,99,100,101,102]. These factors can also have an important influence on disability, independent of their effect on pain [103,104,105,106]. For example, sleep problems are common in OA, with at least 50% of individuals reporting difficulties in initiating or maintaining sleep [19,107]. The interrelations between sleep and pain have been well characterized over the past two decades, with epidemiological, experimental, and clinical research providing broad support for a bidirectional relationship [98]. Consistent with these findings, a number of studies have linked sleep problems with increased pain and pain sensitivity among individuals with OA [108,109,110,111,112], which suggests that sleep could be an important treatment target for reducing OA pain—although clinical trials have not always supported that premise [113]. 

As many as 40% of people with OA have anxiety, depression, or both, as compared to 5–17% in the general population [114]. OA pain and depressive symptoms interact in a recursive cycle, with each contributing to increased fatigue and disability, which may lead to pain worsening over time [20]. Furthermore, in a large sample of pain-free adults, high anxiety was found to predict new onset joint pain over a 12-month follow up period [115] and it was associated with increased pain sensitivity in people with established knee OA [115]. Similarly, people with OA who had the highest levels of psychological distress and pain vigilance showed a generalized increase in pain sensitivity [116], while higher levels of pain catastrophizing have been associated with a long-term worsening of OA pain [117]. 

Conversely, resilience characteristics may offset maladaptive psychosocial factors. For example, increased positive affect in people with knee OA predicts lower joint pain intensity and it is associated with attenuated temporal summation [118], a measure of amplified central nociceptive processing. Similarly, dispositional optimism is associated with lower depression symptoms and greater life satisfaction in people with OA [119], while higher levels of self-efficacy are associated with long-term stability or improvement in OA pain [117]. Finally, more social support has been associated with reduced pain intensity, less distress, and greater activity levels amongst those with chronic pain generally [120,121], and in people with OA specifically [122], while the OA pain and depression symptoms are less strongly correlated in the presence of social support [119]. 

### 1.3. Summary and Aims

OA pain involves a complex interplay of mechanisms, some of which relate to the underlying joint pathology and some of which are distinct—relating to the altered processing and interpretation of nociception in the central nervous system. To date, there are no established disease modifying interventions that can halt or reverse OA related cartilage loss or disease progression. Therefore, treatment is focused on alleviating pain and maintaining or improving physical and psychological function. Rehabilitation is widely recommended as first-line treatment for OA in evidence based clinical guidelines [123,124,125], as it is safer and, in many cases, more effective at reducing pain than the best established pharmacological interventions [126,127]. This paper provides a state-of-the-art overview of rehabilitation interventions for OA pain. In the sections below, we review the best evidence for rehabilitation—including self-management programs, exercise, weight loss, cognitive behavioral therapy, adjunct therapies, and the use of aids and devices. Next, we explore several promising directions for clinical practice, including novel education strategies to target unhelpful illness and treatment beliefs, methods to enhance the efficacy of exercise and innovative, brain-directed treatments. Finally, we discuss potential future research in areas, such as treatment adherence and personalized rehabilitation for OA pain.

## 2. State-of-the-Art Rehabilitation

In this section we focus on synthesizing evidence from international treatment guidelines, meta-analyses, systematic reviews, and, at times, recent randomized controlled trials. A non-systematic search of the literature was performed in PubMed, Scopus, and Google Scholar using the following search terms: rehabilitation, exercise, non-pharmacological, conservative, osteoarthritis, and pain, in order to achieve this. Where appropriate, we used ‘systematic review’, ‘meta-analysis’ and ‘randomized controlled trial’ filters. Additionally, several international treatment guidelines from the last decade were sourced and utilized. 

Importantly, much of the evidence discussed in this section comes from studies in knee OA and, to a lesser extent, hip and hand OA. There may be key differences in the optimum rehabilitation strategies that were employed according to the joint(s) affected by OA. Where possible, we attempt to provide examples where the strength of evidence or recommendations differ according to the joint involved. However, until such time, as a sufficient number of high-quality studies are performed in people suffering from OA at other joints, some extrapolation from the available literature is necessary. 

Furthermore, it is important to emphasize that the overall quality of the evidence considerably varies across interventions (Table 1). Where possible, we attempt to highlight the quality of the evidence in each section, as indicated in recent systematic reviews and international treatment guidelines. Finally, the nature of many of the treatments discussed means that the effective blinding of the intervention is difficult or, in some cases, impossible to achieve. Thus, as with any intervention, part of the therapeutic effect described is likely to be non-specific in nature. 

### 2.1. Self-Management Programs

Given that OA is a chronic disease, its symptoms require long-term, habitual management. A passive coping style, through which people become behaviorally inhibited and avoid taking an active role in self-managing their pain, has been consistently related to poorer outcomes across various chronic pain disorders [128,129,130], including OA [131,132]. Several OA treatment guidelines recommend self-management interventions as a core component in the effective management of OA [123,133,134]. The notion that expectancy, belief, and motivation shape the pain experience and the accompanying behaviors that contribute to either chronic pain adaptation or disability is critical to the concept of self-management. Self-management interventions are programs that aim to teach people to take an active role in managing their condition through any combination of education, behavior change, and psychosocial coping skills [135]. For example, these interventions can include modules providing information regarding the health condition, healthcare resource utilization, stress management techniques, physical exercises, and interpersonal problem-solving skills. These programs can be heterogeneous in the implementation of specific strategies, but commonly try to counter unhelpful illness and treatment beliefs and impart transferable skills that empower individuals to effectively manage their symptoms long-term [136]. 

The existing evidence for self-management program efficacy in OA shows mixed outcomes. A meta-analysis pooling results from 13 trials found an overall small beneficial effect of self-management programs on pain reduction, but no significant impact on quality of life or physical function [137]. However, the authors found a specific pooled effect of pain reduction and quality of life improvement for self-management programs that contained exercise programs, which suggests the latter might be a key component for OA. Furthermore, Kroon and colleagues examined 29 studies comparing education-specific self-management interventions to other interventions for adults with OA. The authors found overall weak effect sizes for self-management programs that are focused on disease education over other treatments [138], which suggests that current education strategies may be suboptimal. Both reviews concluded that self-management programs vary widely in their content (e.g., focus on managing OA symptoms vs. holistic well-being), duration (e.g., single session vs. ongoing, weekly vs. monthly), and method of delivery (e.g., in-person vs. telehealth, individual vs. group, lay leader vs. healthcare professional), limiting conclusive evidence for their efficacy. 

### 2.2. Exercise 

Regular exercise is considered to be a core treatment for OA and it is universally recommended amongst treatment guidelines for all individuals with OA, regardless of their individual presentation [126,139,140]. Exercise has a number of potential benefits, including improving pain [126,141,142], physical function [143], and mood [144], as well as decreasing the risk of secondary health problems, including cardiovascular, metabolic, neurodegenerative, and bone disorders [145]. Exercise likely reduces OA pain by several different mechanisms, including increased central nervous system inhibition [146,147], local [148] and systemic [149] reductions in inflammation, psychosocial effects [150], and biomechanical effects at the affected joint [151]. 

Exercise for OA might include low impact aerobic exercise, such as walking or cycling, resistance training for muscle strengthening, stretching, and other forms of exercise, such as Tai Chi or Yoga. Importantly, exercise has very few adverse effects [152], does not appear to accelerate joint degeneration [152,153,154], and has similar or better effect sizes for OA pain as compared to commonly used analgesics, such as acetaminophen and non-steroidal anti-inflammatory drugs (NSAIDS) [126]. 

At this time, there is insufficient evidence to determine whether one type of exercise is superior, with systematic reviews suggesting that several different types of exercise are effective for OA [125,126,127,155]. Resistance training is the most studied type of exercise for individuals with OA. The strongest evidence for pain relief and improvements in function exists in people with knee and hip OA [141,142], with fewer high quality studies in hand OA [156]. Resistance training interventions can be home or clinic based, and should be undertaken for at least 2–4 months in order to maximize the clinical benefits [157]. Aerobic exercise is widely recommended in treatment guidelines [123,124,126,133,134,158,159], effectively relieves OA pain [127], and it may have additional benefits, such as promoting cardiovascular health and weight loss. However, the majority of RCTs that were conducted in OA populations include interventions that are not solely aerobic but have elements of strengthening and stretching to varying degrees [158]. Hence, their findings could be viewed as supporting the inclusion of aerobic exercise within a wider program of exercise. Other forms of exercise, such as tai chi, yoga, and whole body vibration, currently have less evidence to support them, with low to very low quality evidence that shows both positive and negative effects [125,160]. Some guidelines [124,126] have made conditional recommendations concerning land-based versus water-based interventions. Overall, there is greater support for land-based exercise that is based upon both the magnitude of effects in RCTs and the quality of evidence [125]. However, some individuals with hip or knee OA might be better suited or have a preference for water-based exercise for some parts of their rehabilitation program [124]. 

For exercise to be most successful, it must be of sufficient volume to elicit adaptations that relieve pain and improve physical function. Concerning resistance training, guidelines [157] highlight that pain relief can occur, irrespective of the equipment (dynamometers, weights, bands) utilized, the type of exercise (e.g., isokinetic, isotonic), and the muscle action (i.e., isometric, eccentric concentric) performed. Despite such a range of options, consideration should be given within the overall program to those exercises that more closely simulate the type of muscle activity utilized in the work tasks and/or activities that are required in the individual’s daily life. Other parameters, such as the load, the number of repetitions within a set, the number of sets performed per session, the rest intervals between sets, and the frequency of sessions per week should all be carefully considered. For aerobic exercise, the intensity, type of exercise, how it is performed (e.g., continuous or in intervals), and frequency per week are all important. For aerobic and strengthening exercises, the suggested starting points for these training parameters are well described in the ACSM public health guidelines [161], with a recent clinical guideline [162] recommending these levels of exercise are embedded within standard care in people with OA. Specifically, a minimum of 150 min. moderate intensity or 75 min. vigorous intensity aerobic exercise per week is recommended (in bouts of at least 10 min). For resistance training, two sessions per week, with two sets of eight to 12 repetitions at a load of 60% to 70% of one repetition maximum can be recommended as a starting point [161]. A rest period of ≥48 h between resistance training sessions is suggested in order to optimize muscle hypertrophy [161].

The need for personalized, individually tailored exercise programs while taking into account a person’s exercise preferences has been highlighted [124,163], as these are considered more likely to achieve long term exercise adherence [164]. Another key point concerning adherence is that education is often needed to emphasize that appropriate levels of exercise are safe, and while pain exacerbations may occur at times, these will reduce over the course of a training program [165] and people with OA will continue to benefit from ongoing exercise. It is recommended [162] that exercise regimes be provided by health care professionals with suitable backgrounds (e.g., physiotherapists) who regularly reassess progress and modify training parameters to limit and manage symptom exacerbations. The importance of progression has also been highlighted by Brosseau et al. [157] and Magni et al. [156], who noted that the majority of trials that did not find positive results for an exercise intervention had not implemented a program that continually reassessed and progressed the training volume over time. 

Concerning the overall duration of the exercise program, one could argue that exercise should be considered as a lifestyle change and undertaken perennially. To date, most RCTs of exercise interventions involving people with OA have been undertaken over a two to four month period with few interventions extending past six months. While benefits can continue for several months, it is well known from the literature related to athletic training that when training is stopped completely, a detraining effect be observed within two to three weeks [166], and this decline steadily continues, depending upon factors, such as age and physical activity level. Importantly, there is evidence [166] that these declines in performance can be slowed with booster sessions that are undertaken less regularly (e.g., one training sessions every one to two weeks for resistance and aerobic exercise). Booster sessions may also be useful in promoting longer-term exercise adherence [167]. 

### 2.3. Weight Loss

Increased body weight is considered to be an important modifiable risk factor for the onset and progression of pain and radiographic findings [168,169,170] of OA, specifically at the knee and hip [124,171,172,173]. In symptomatic knee OA, this risk is doubled with every 3–4 kg/m^2^ increase in Body Mass Index (BMI) [168]. Furthermore, obesity is associated with a systemic pro-inflammatory state that may accelerate joint degeneration [174] and increase the sensitization of the nociceptive system, thereby enhancing OA pain [175]. As a result, weight loss interventions are recommended by several international treatment guidelines for OA as part of the core treatment for people with knee and/or hip OA that are overweight or obese [123,124,126,133,134,140,159,169,176,177,178,179,180]. Furthermore, education regarding the importance of maintaining a healthy lifestyle and body weight is recommended for all people with OA [124]. 

Although a consensus regarding the BMI cut off for determining the target population for weight loss programs is lacking [180], weight loss is typically recommended in individuals presenting with symptomatic OA and a BMI ≥ 25 kg/m^2^ [124,133,169,177] (pre-obesity BMI value, as defined by the World Health Organization [181]). Care should be taken when advising weight loss to older people (aged > 65 years) to ensure the maintenance of lean body mass and bone density [124,182].

The importance of weight loss programs is supported by moderate to high quality evidence reporting improvements in pain and disability after weight loss in people with knee OA [177,183,184,185]. More recently, a reduction in systemic inflammatory biomarkers has also been observed [186]. Ideally, weight loss interventions should comprise a combination of dietary advice and exercise [124,177,184,187,188,189,190], including explicit individual weight loss goals and problem solving regarding how to reach these goals [159,184,191,192]. The benefits of weight loss interventions are dose dependent—with higher amounts of weight loss resulting in larger benefits—starting at a minimum of 5–7.5% body weight loss [124,184,185,193]. 

Although the evidence for weight management programs is generally limited to knee OA, the systemic health benefits of weight loss and maintaining a healthy body weight are not negligible. Therefore, weight loss principles are most likely transferable to people with hip OA [134], in which being overweight is known to be a risk factor [124,171], and possibly also to individuals with OA in other joints [123,176].

### 2.4. Cognitive Behavioral Therapy

Cognitive behavioral therapy (CBT) is increasingly recognized as a valuable intervention for OA pain. A recent guideline [124] recommends CBT for selected people with knee and/or hip OA, particularly those with psychosocial comorbidities. Another recent treatment guideline specifically focused on arthritis pain management [125] concluded there is now moderate evidence supporting CBT for OA pain and recommended that appropriately selected individuals should receive both psychological and sleep interventions. CBT for pain generally involves the identification and facilitation of individually specific behavioral goals that promote activity and social engagement while minimizing withdrawal and guarding. Cognitive barriers to engagement in adaptive behaviors (e.g., pain catastrophizing) are also identified and systematically challenged in the course of CBT. While CBT for pain has been extensively investigated in a variety of chronic pain disorders, relatively fewer trials are available for OA [194]. A recent systematic review and meta-analysis identified 12 RCTs that examine psychological interventions in an OA population [195]. While heterogeneous in the focus of their treatment, overall, these studies demonstrated small reductions in pain and fatigue and moderate to large improvements in self efficacy and pain coping. Interestingly, despite focusing on non-pain symptoms, several RCTs that involve CBT interventions have also demonstrated modest efficacy for improving OA pain. For example, an online CBT intervention for depression in people with knee OA and comorbid depression significantly reduced depression symptoms, but also demonstrated a medium-sized effect on OA pain and function relative to treatment as usual [196]. Similarly, CBT for insomnia has been tested in several different cohorts of people with knee OA and comorbid insomnia. Smith et al. [197] demonstrated that CBT for insomnia improved wake after sleep onset—a key marker of sleep disruption—among people with OA. Statistically significant reductions in pain severity were observed through six-month follow-up, and improvements in wake after sleep onset predicted reduced pain at follow-up. Small to medium sized effects on pain were observed in another RCT of CBT for insomnia in people with knee OA and comorbid insomnia [198], as well as a larger population-based RCT of CBT for insomnia in people with knee OA recruited from primary care clinics [199]. Finally, Vitiello et al. [200] conducted a three-arm trial comparing CBT for pain with CBT for comorbid pain and insomnia symptoms and an education control in people with OA and comorbid insomnia. Interestingly, the mean pain levels were not significantly improved in any treatment condition by post intervention. However, in subgroup analyses, those individuals who had clinically meaningful improvements in insomnia symptoms (≥30% reduction) by post-intervention (two months) demonstrated significant long-term reductions in pain at both nine and 18-month follow-up. Thus, there is growing evidence that CBT interventions, whether directed at pain or at other problems, such as depression or sleep, can produce clinical benefits and should be considered in appropriate individuals. 

### 2.5. Adjunct Treatments 

Several OA treatment guidelines include recommendations regarding the use of adjunct treatments, such as manual therapy, thermal modalities, acupuncture, and electrotherapies [123,124,126,133]. The quality of evidence used in forming these recommendations is generally low quality, with a high risk of bias, and it is apparent that more studies involving these interventions are needed before strong conclusions can be drawn. Manual therapy (that may include joint mobilization/manipulation and massage) is generally not recommended as a stand-alone treatment [123,124,133,178], but a short course of manual therapy [124], provided as an adjunct treatment to facilitate engagement in active strategies, such as exercise, is recommended by several guidelines [123,124,178] and might enhance pain relief compared to exercise alone [201]. 

The use of thermal modalities, such as superficial heat or cold, may provide short-term relief of symptoms and are low cost, low risk interventions that can be incorporated into self-management, and are therefore widely recommended as adjunct treatments in several guidelines [123,124,178], despite low quality evidence supporting their use. 

Some treatment guidelines recommend acupuncture, particularly for knee OA [178], but others recommend against it [123,124,133], as well conducted systematic reviews have typically shown that the benefits as compared to placebo are small and of questionable clinical importance [202,203]. There may be value over usual care for individuals with OA who have positive treatment expectations regarding acupuncture, although much of the clinical benefit may be due to non-specific effects [204]. 

The evidence for electrotherapies is mixed and it is limited by low quality studies with short follow up times. A recent systematic review [205] suggests that interferential and high frequency TENS may have some benefit, while evidence is generally lacking for other modalities. Several treatment guidelines recommend using TENS as an adjunct treatment [123,124,163,178]. The portability of TENS units, their relatively low cost, and their ability to be used at home as part of a self-management strategy or in combination with exercise makes this form of electrotherapy particularly attractive. 

### 2.6. Aids and Devices

A large range of braces, insoles, and splints are available and marketed for individuals with OA. In general, these devices are designed to produce mechanical effects that decrease load on the OA affected joint or offer additional sensory input that may enhance proprioception and joint stability. There is generally low to very low evidence supporting their use and very little information that can be used clinically to determine which device may or may not be appropriate for a given individual [164]. 

Unloading braces have metal and soft materials, which are located or positioned to reduce joint forces during gait activities, particularly in knee OA. A recent treatment guideline [124] recommended that valgus unloading braces should not be offered for medial compartment OA. For lateral compartment OA, a decision to recommend either for or against the use of a varus unloading brace could not be made. These findings were similar to other treatment guidelines [123,125], where low quality evidence provided unclear to positive support of braces. Individuals with lower limb musculoskeletal conditions have long used canes (walking sticks). Based on low and very low evidence levels, these devices may be useful for some individuals with knee and/or hip OA, and they were conditionally recommended by some treatment guidelines [123,124].

For other types of support (e.g., elastic bandages/soft braces/tape) that are thought to primarily improve joint proprioception, a EULAR guidelines group [125] indicated that while there was low quality evidence, it was generally positive. With hand OA, splints are often utilized to manage pain, alignment, and instability issues, particularly at the thumb. Long term use (>3 months) of splints has been recommended for pain that is associated with thumb OA [163,206], with short term use and use in other joints considered to be ineffective [125,206]. Overall, the evidence supporting these recommendations is considered low quality.

Regarding footwear for lower limb OA, a recent treatment guideline [124] has recommended that unloading shoes, minimalist footwear, and rocker-sole shoes should not be offered to individuals with knee OA at this time, due to limited evidence supporting their efficacy. It was also thought that high heel shoes should be avoided but that shoes with additional shock absorbing properties might be suggested to some people. The support for shoe inserts is mixed, with recent treatment guidelines [124,125], finding low to very low evidence supporting lateral wedge shoe insoles for medial compartment OA and medial wedge insoles for lateral compartment OA with benefits ranging from unclear to positive.

## 3. Promising Directions for Clinical Practice

### 3.1. Improved Education Strategies to Address Maladaptive Pain-Related Beliefs

People with OA can display fear avoidance behaviors [16,17,18] that limit their engagement in effective rehabilitation strategies, such as regular exercise. A recent Cochrane review [207] has highlighted many of the unhelpful beliefs held by people with hip and knee OA that help to shape these behaviors. Many people describe being confused about the cause of their pain and bewildered by its variability and unpredictable nature [207]. Furthermore, as movement frequently increased their pain, they worried that this might be doing their joint further harm and described avoiding physical activity and exercise as a result [207]. These findings suggest a need for strategies tackling these maladaptive beliefs and behaviors.

Pain neuroscience education is a cognitive-based intervention that is aimed at reconceptualizing pain by de-emphasizing pathoanatomical content and focusing on other factors, such as the discordance between imaging findings and pain, peripheral and central sensitization, cognition, mood and lifestyle factors that may contribute to the development and persistence of pain, all within a biopsychosocial framework [208,209]. The use of this educational strategy in people with chronic pain in order to change pain related beliefs, improve health behaviors and—importantly—desensitize the central nervous system, is supported by high quality evidence [209,210,211]. Although pain neuroscience education has been studied in several chronic pain populations [211], the evidence in people with OA is still limited and mainly focused on people undergoing knee arthroplasty [212,213]. In these studies, positive effects of preoperative pain neuroscience education were found in terms of psychosocial measures (pain catastrophizing [212] and kinesiophobia [212,213]), pressure pain thresholds [213], and peoples’ beliefs regarding their scheduled surgery [213].

As the evidence suggests that current education strategies for OA have limited success [138], a pain neuroscience approach might provide an alternative and more effective means of targeting unhelpful illness and treatment beliefs, particularly at the beginning of a rehabilitation program. This may help to reduce pain and psychological distress as well as facilitate engagement and adherence in exercise-based interventions. Such an approach might be particularly effective if the pain-relieving effects of exercise and its role in desensitizing the nociceptive system are specifically emphasized and incorporated into the education session(s), as this has been shown to enhance positive expectations and increase exercise induced pain relief [214].

### 3.2. Enhancing the Effectiveness of Resistance Training

The weakness of muscles adjacent to the painful joint(s) is a common feature of OA. Adequate muscle strength is required for many activities of daily living [215,216] and muscle weakness is a major factor contributing to OA related functional disability [217,218]. In the lower limb, muscles may also have a protective role, attenuating mechanical loading of the OA joint during gait and other activities [27,219]. There is some evidence that higher quadriceps muscle volume might protect against incident knee pain and ongoing cartilage loss [29,30] and recent findings suggest that the magnitude of quadriceps strength gains partially mediate the pain-relieving effect of resistance training in knee OA [220]. Unfortunately, muscle strength gains during resistance training are often compromised by arthrogenic muscle inhibition, an ongoing neural inhibition of muscle activation due to factors, such as joint effusion, nociception, and sensory loss [23,221,222,223]. This problem is widely recognized in knee OA [224], although arthrogenic muscle inhibition might also contribute to ongoing muscle weakness at other joints [225,226,227,228]. Importantly, it has been shown in people with OA that adjunct disinhibitory interventions, such as cryotherapy, TENS, and NSAIDS, can be used to reduce arthrogenic muscle inhibition [229,230] and, when used in conjunction with resistance training, might enhance muscle strength gains when compared to resistance training alone [230,231,232].

Another promising intervention that might enhance the efficacy of resistance training in OA is blood flow restriction training. Blood flow restriction training utilizes an inflatable cuff or band to partially occlude blood flow in the exercising muscles. Importantly, blood flow restriction allows exercise of very low load (e.g., 20–40% of 1RM) to produce significant gains in muscle strength and size [233], seemingly due to exaggerated metabolic stress when training the muscle(s) under partial occlusion. To date, blood flow restriction training has been largely applied in healthy populations [233], but it is an attractive intervention for OA, as it has the potential to accelerate muscle hypertrophy and strength gains while also notably reducing the mechanical load placed on the affected joint(s) during training—thus potentially minimizing exercise-induced flares in joint pain. Preliminary evidence in populations that are relevant to OA suggests that blood flow restriction training is associated with less pain during exercise [234,235,236] and may produce similar [234,235,236,237] or greater [238] gains in muscle strength than resistance training performed without blood flow restriction.

### 3.3. Brain Directed Treatments of Sensorimotor Networks

There is growing evidence of dysfunction in brain sensorimotor networks amongst people with OA, with observations of widespread tactile hypoaesthesia [239], reduced tactile acuity [240], body size distortions [41], neglect-like symptoms [42], altered primary somatosensory cortex volume [96], impaired implicit motor imagery performance [42,241], and both disinhibition [242] and reorganization [243] of the primary motor cortex. While the clinical implications of these changes are yet to be fully elucidated, there is evidence that some might be important treatment targets for OA pain. For example, recent RCTs provide preliminary evidence that non-invasive brain stimulation of the primary motor cortex may reduce OA pain, either when delivered alone or in combination with other interventions [244,245,246,247]. Furthermore, in other chronic pain conditions, sensory discrimination training has been used to reverse deficits in tactile acuity that are similar to those that were observed in OA [248,249]. These interventions have also been shown to reduce chronic pain intensity [248,249,250,251], with the magnitude of pain relief being strongly correlated to improvements in tactile acuity [249,250] and cortical sensory representation of the affected body part [250]. Finally, it has been shown that presenting a multisensory illusion of the painful OA joint(s) stretching or shrinking can produce immediate and, in some cases, substantial pain relief [40,252], and that this intervention can partially correct distorted size perceptions of the OA affected limb [41]. While still in its infancy, the use of brain directed treatments that target impaired sensorimotor networks is a promising clinical direction that might have important future implications in the rehabilitation of OA pain.

## 4. Promising Directions for Research

### 4.1. Personalized Treatment

In recent years, a number of studies have attempted to identify several distinct OA pain phenotypes or subgroups from a larger population [253,254,255,256,257,258]. The potential benefits of such an approach include the identification of key prognostic factors that predict treatment response and, ultimately, the development of more targeted treatments that are personalized to the individual and their dominant pain mechanism(s). For example, individuals with OA who have more pronounced central sensitization, as evidenced by increased temporal summation [259,260,261,262,263,264] and, in some cases, widespread pain sensitivity [265] and reduced conditioned pain modulation [261,266] may experience less pain relief and are at higher risk of persistent pain after peripherally targeted treatments, such as NSAIDs or total joint replacement surgery.

The identification of relevant OA pain phenotypes may also lead to improved rehabilitation strategies. For example, Fingleton et al. [267] have recently shown that the initial response to exercise in people with knee OA varies according to the baseline function of their endogenous descending pain inhibitory/facilitatory pathways—i.e., those with deficient conditioned pain modulation tended to experience increased pain after both aerobic and resistance exercise, while pain was reduced in those with intact conditioned pain modulation and in healthy controls. Studies such as this that explore within-group differences in OA may allow for better identification of likely non-responders to treatment and facilitate the development of alternative, more personalized strategies (e.g., the combination of centrally acting analgesics with exercise) to enhance clinical outcomes in these individuals.

Similarly, despite consistent evidence of the positive treatment effects of psychological therapies for OA, there is a gap between the strength of evidence for process-oriented measures and core outcomes. For example, whereas psychological therapies produce large effects on active coping, the effects on pain and function tend to be smaller [268]. One reason for this discrepancy might be the fact that a portion of people with OA have more pronounced central sensitization [89], which may be less responsive to traditional CBT and related psychosocial therapies [269]. Lumley & Schubiner [269] have recently proposed a novel treatment paradigm that is intended to target people with central sensitization by performing a detailed intake assessment and tailoring treatment with pain neuroscience education, cognitive therapy, mindfulness, behavioral desensitization, emotional expression, and interpersonal communication skills. An initial RCT in fibromyalgia patients [270] showed that this treatment approach outperformed traditional CBT in lowering the fibromyalgia symptoms and widespread pain. Future work is needed to similarly evaluate the differential efficacy between this novel pain treatment and traditional CBT for people with OA subtyped based on their degree of central sensitization.

### 4.2. New Ways of Understanding and Enhancing Treatment Adherence

Better adherence to rehabilitation interventions in OA is typically associated with greater symptom improvement [196,271,272,273]. However, previous work has documented poor to adequate adherence among this population [274,275,276,277]. Furthermore, current strategies that aimed at increasing adherence are not uniformly effective. A meta-analysis of adherence interventions found significant improvements in only 18 of the 42 included trials [278]. This low efficacy rate emphasizes the need for novel, more potent interventions to promote adherence to rehabilitation programs. The predictors and mechanisms of adherence to rehabilitation interventions in OA are not fully established. However, previous work suggests that motivation is a consistent predictor of treatment adherence in OA and across chronic pain conditions [279,280,281,282,283,284].

Although psychosocial factors predicting treatment motivation amongst those with chronic pain have been probed, physiological mechanisms potentially subserving these processes have not been examined. For example, a potentially important physiological mechanism of treatment motivation is mesocorticolimbic function [285]. This neural system—colloquially referred to as “the reward system”—has been extensively associated with motivational processes and reward learning in humans and animal models [286,287,288,289]. On behavioral tasks, individuals with chronic pain demonstrated altered reward learning [290,291,292] and reduced motivation [280,293] when compared to individuals without pain. Factors, such as depression and opioid use, are likely to further compound these effects. Neuroimaging data support these findings by demonstrating aberrant mesocorticolimbic system structure [96,294] and function during pain relief—potentially reflective of attenuated rewarding effects of analgesia [295,296,297,298,299]—and wakeful rest [300,301,302] among people with chronic pain.

It is possible that altered reward learning and attenuated rewarding effects of pain relief contribute to reductions in treatment motivation and the resultant adherence to rehabilitation. Future studies might examine the extent of the relationships among baseline treatment motivation, mesocorticolimbic function, and engagement in adaptive rehabilitation strategies, such as regular exercise. This question could be addressed while using a combination of neuroimaging, questionnaire, and ecological momentary assessment (e.g., daily diary) data. Such an approach might inform the development of adjunct interventions to bolster the rewarding effects of pain relief early in the course of rehabilitation (e.g., via non-invasive brain stimulation, endogenous reward system training) and promote better long-term adherence to rehabilitation interventions.

## 5. Conclusions

Treatment strategies for OA pain should be broadened beyond a simple focus on the affected joint(s). While joint directed treatments remain sensible, it is important to screen for, recognize, and appropriately manage other factors (e.g., central sensitization, psychosocial factors, sleep problems) that may be contributing to an individual’s pain experience. Rehabilitation is considered first line treatment for OA pain. Core interventions that are widely recommended by evidence-based OA treatment guidelines include regular aerobic and resistance exercise, self-management programs, and, where appropriate, weight loss. A range of other options, such as manual therapy, thermal modalities, TENS, and joint braces/splints, may also be useful adjunct therapies, although there is currently less evidence supporting their use. CBT is increasingly recognized as a valuable treatment option for selected individuals and may have important clinical benefits for psychological and sleep related comorbidities. While further evidence is needed to support their clinical utility, novel treatment approaches, such as pain neuroscience education, the use of disinhibitory interventions to augment resistance training, blood flow restriction training, and brain directed treatments (e.g., illusory resizing and non-invasive brain stimulation) may play an important role in the future rehabilitation of OA pain. In addition, key avenues for future research include the development of personalized rehabilitation interventions and improved methods to both enhance treatment adherence and better understand its physiological underpinnings.

## Figures and Tables

**Table 1:** Best evidence table for the rehabilitation of people with osteoarthritis: Summary of the evidence and recommendations for practice.

Intervention	LoE	Summary of Evidence	Main Profession(s) Involved	Recommended by Recent Treatment Guidelines			Authors’ Recommendations for Practice
				Yes [Ref]	No [Ref]	Uncertain/Not Included [Ref]	
Self-Management Programs							
	1A	Low to moderate quality evidence of no or small positive effect on pain, function and quality of life. Large degree of variability in delivery. Self-management programs that include exercise may be more effective.	General practitioner OR psychologist OR physiotherapist OR internet based	ACR [178] EULAR knee & hip [159] NICE [164] OARSI knee [126] PANLAR [163] EULAR hand [207] EULAR pain [125]	-	RACGP [124]	Provide education to enhance understanding of OA and its treatment in order to counter misconceptions that OA inevitably progresses, cannot be treated and that symptoms are closely related to imaging findings. Encourage active self-management and positive behavioral changes such as regular exercise, weight loss, good sleep hygiene and activity pacing. Avoid use of language such as “wear and tear” and “bone on bone” as this may perpetuate unhelpful illness and treatment beliefs.
Exercise							
	1A	Moderate to high quality evidence of a positive effect on pain and function in hip and knee OA that is sustained for several months. Low quality evidence for hand OA. Several different types of exercise may be effective (not clear which is best). Programs that progress exercise volume over time and deliver higher overall doses of exercise may be more effective.	Physiotherapist OR exercise physiologist	ACR [178] EULAR knee & hip [159] NICE [164] OARSI knee [126] PANLAR [163] EULAR hand [207] EULAR pain [125] RACGP [124]	-	-	A combination of resistance training and low impact aerobic exercise should be provided and tailored to the impairments, functional requirements and preferences of the individual. Consider water-based exercise in individuals who prefer it, or cannot tolerate land-based exercise. Provide education that exercise is safe and provide reassurance about potential symptom exacerbations. Exercise should continue for at least 8 weeks and training volume progressed regularly in order to maximize benefits. Provide booster sessions to minimize detraining effects and enhance long term exercise adherence.
Weight Loss							
	1A	Moderate to high quality evidence of dose dependent improvements in pain and function with weight loss in knee OA, with presumed benefits for hip OA and possibly other joints.	Dietician OR physiotherapist OR psychologist OR general practitioner	ACR [178] EULAR knee & hip [159] NICE [164] OARSI knee [126] PANLAR [163] EULAR pain [125] RACGP [124]	-	EULAR hand [207]	Target a minimum weight loss of 5–7.5% of body weight in all people with knee and/or hip OA who have a BMI of ≥ 25 kg/m^2^. Greater weight loss will likely result in increased benefit. Provide education about the importance of maintaining a healthy body weight to people with a BMI of < 25 kg/m^2^. Explicit weight loss goals and problem solving on how to achieve those goals should be planned in a patient centered, collaborative manner. Consider a combination of individualized strategies such as regular weight monitoring, increased physical activity, social support, meal plans, limiting portion size, reducing fat and sugar intake, time restricted feeding and addressing behavioral triggers to eating (e.g., stress, poor sleep). Combining weight loss with regular exercise will increase its benefits.
Cognitive Behavioral Therapy							
	1A	Low to moderate quality evidence that CBT has small positive effects on OA pain and fatigue and moderate to large positive effects on self efficacy and pain coping. Overall mixed (no to medium positive) effects on pain in trials of CBT for insomnia.	Psychologist OR internet based	ACR [178] EULAR pain [125] RACGP [124]	-	EULAR hand [207] PANLAR [163] OARSI knee [126] NICE [164] EULAR knee & hip [159]	Consider CBT if significant psychosocial comorbidities (e.g., depression) exist that may interfere with effective pain management and rehabilitation. Consider CBT based sleep interventions for people with OA who have co-morbid sleep problems. Consider CBT if psychological factors such as fear of movement and pain catastrophizing are a barrier to physical function and engaging in exercise.
Adjunct Treatments							
Manual therapy	1A	Low quality evidence that manual therapy alone or in combination with exercise has short term positive effects on pain and function	Physiotherapist OR osteopath OR chiropractor	RACGP knee & hip [124] ACR [178] NICE [164] (all as an adjunct treatment only)		EULAR knee & hip [159] OARSI knee [126] PANLAR EULAR hand [207] EULAR pain [125]	Consider providing a time-limited course of manual therapy as an adjunct to exercise if the individual finds it beneficial.
Thermal modalities	1B	Low quality evidence that thermal modalities may provide short term positive effects on pain	Self-management OR physiotherapist OR general practitioner	PANLAR [163] (stand-alone)RACGP knee & hip [124] ACR [178] NICE [164] (all as an adjunct only)		EULAR knee & hip [159] EULAR hand [207] EULAR pain [125]	Consider the use of local hot or cold packs as a pain self-management strategy or as an adjunct to exercise if the individual finds this beneficial.
Acupuncture	1A	Low to moderate quality evidence of mixed (no to medium positive) effects on pain compared to sham acupuncture interventions.	Acupuncturist	ACR [178]	NICE [164] RACGP knee & hip [124]	EULAR knee & hip [159] OARSI knee [126] PANLAR [163] EULAR hand [207] EULAR pain [125]	Acupuncture is generally not recommended for the treatment of OA. Consider a time-limited course of acupuncture as an adjunct treatment only if the individual has positive treatment expectations.
Electro-therapy	1A	Low quality evidence that interferential and high-frequency TENS may have positive effects on pain and function. Limited evidence for other forms of electrotherapy.	Self-management OR physiotherapist OR general practitioner	ACR [178] NICE [164] PANLAR [163] RACGP knee & hip [124]		EULAR knee & hip [159] OARSI knee [126] EULAR hand [207] EULAR pain [125]	Consider the use of TENS as a pain self-management strategy or as an adjunct to exercise if the individual finds this beneficial.
Aids and Devices							
Unloading braces	1B	Limited, low quality evidence that unloading braces have no effect on pain or function in knee OA	Not discipline specific	NICE [164] OARSI knee [126]	RACGP knee & hip [124]	ACR [178] EULAR knee & hip [159] EULAR hand [207]	Unloading braces are not recommended in the treatment of OA.
Cane (walking stick)	2B	Limited, low quality evidence that the use of a cane has positive effects on pain and function in knee OA with possible benefits for other lower limb joints.	Not discipline specific	ACR [178] EULAR knee & hip [159] EULAR pain [125] RACGP knee & hip [124]		EULAR hand [207]	Consider the use of a cane (walking stick) in people with lower limb OA if the individual finds this beneficial or has notable problems with balance and mobility. The cane should be used on the contralateral side and adjusted to the height of the greater trochanter.
Soft braces	1A	Low quality evidence that soft braces have mixed (no to positive) effects on pain and function in knee OA	Not discipline specific	NICE [164] OARSI knee [126] PANLAR [163] EULAR pain [125]		ACR [178] EULAR knee & hip [159] EULAR hand [207]	Consider the use of a soft brace (e.g., neoprene brace, elastic sleeve) in people with knee OA if the individual finds this beneficial.
Splints	1A	Low quality evidence that long term use of splints have a positive effect on pain and function for thumb OA only. Limited evidence that one type of splint is better than any other.	Physiotherapist OR occupational therapist	ACR [178] NICE [164] EULAR hand [207] PANLAR [163] EULAR pain [125]		EULAR knee & hip [159] RACGP knee & hip [124] OARSI knee [126]	Consider the long term use (>3 months) of a splint for people with thumb OA only.
Footwear	1B	Limited, low quality evidence that specific footwear has no benefit on pain and function compared to standard footwear.	Not discipline specific	EULAR knee & hip [159] NICE [164] EULAR pain [125] OARSI knee [126]	RACGP knee & hip [124]	EULAR hand [207] ACR [178] PANLAR [163]	Specific footwear (e.g., minimalist footwear, rocker-sole shoes, unloading shoes) is not recommended in the treatment of OA. Consider the use of shoes with appropriate shock absorbing properties for people with lower limb OA.
Shoe Insoles	1B	Low quality evidence that medial wedge shoe insoles may provide positive effects on pain and function for lateral compartment knee OA. Low quality evidence that lateral wedge insoles for medial compartment knee OA have mixed (no to positive) effects on pain and function	Podiatrist OR physiotherapist	ACR [178] NICE [164] OARSI knee [126] PANLAR [163]	RACGP knee & hip [124]	EULAR knee & hip [159] EULAR hand [207]	Consider the use of medial wedge shoe insole for people with lateral compartment knee OA if the individual finds this beneficial. Lateral wedge shoe insoles are not recommended in the treatment of knee OA.

Level of Evidence (LoE): 1A: Systematic review of randomized controlled trials; 1B: Individual randomized controlled trials; 2A: Systematic review of cohort studies; 2B: Individual cohort study or low quality randomized controlled trials; 3A: Systematic review of case-control studies; 3B: individual case-control study; 4: Case-series; 5: Expert opinion. Quality of evidence is as reported in recent systematic reviews or international treatment guidelines. Abbreviations: LoE = Level of evidence; OA = osteoarthritis; Ref = Reference; CBT = Cognitive behavioral therapy.
